# Evaluation of the Cytotoxic and Apoptotic Activities and the Phytochemical Profile of *Euphorbia indivisa* (Engelm) Tidestr.

**DOI:** 10.3390/ijms27219858

**Published:** 2026-11-03

**Authors:** Mónica I. Ramos-Adame, Paola Maycotte, Fabiola L. Sarmiento-Salinas, Mónica Morales-Aguilar, Alexandre T. Cardoso-Taketa, A. Berenice Aguilar-Guadarrama

**Affiliations:** 1 Centro de Investigaciones Químicas, IICBA, Universidad Autónoma del Estado de Morelos, Av. Universidad 1001, Col. Chamilpa, Cuernavaca 62209, Morelos, Mexico; monica.ramos@uaem.edu.mx; 2 Centro de Investigación Biomédica de Oriente, Instituto Mexicano del Seguro Social, Carretera Federal Atlixco-Metepec, Metepec 74360, Puebla, Mexico; bisbenzimida@gmail.com (P.M.); fabiola.sarmiento@udlap.mx (F.L.S.-S.); 3 Secretaría de Ciencia, Humanidades, Tecnología e Innovación (SECIHTI), Mexico City 03940, Mexico; 4 Centro de Investigación en Biotecnología, Universidad Autónoma del Estado de Morelos, Av. Universidad 1001, Col. Chamilpa, Cuernavaca 62209, Morelos, Mexico; moraaguifcb@gmail.com (M.M.-A.); ataketa@uaem.mx (A.T.C.-T.)

**Keywords:** *Euphorbia indivisa*, phytoconstituents, cytotoxicity, apoptosis

## Abstract

Cancer remains one of the leading causes of death worldwide, highlighting the need to develop new therapeutic strategies. In this context, several species of the Euphorbiaceae family have demonstrated cytotoxic activity. The present study evaluated the cytotoxic effect of *Euphorbia indivisa* on three cancer cell lines (Breast cancer, MCF-7; Human pancreatic ductal adenocarcinoma, PANC-1; Human cervical, SiHa), as well as on a nontumorigenic cell line (Immortalized human keratinocyte, HaCaT). The chemical profile of the species was also determined. The hydroalcoholic extract (HAE) and fractions were obtained by maceration and liquid–liquid partitioning using dichloromethane (DCF), ethyl acetate (EAF), and butanol (BuF). Cell confluence and death were determined by analysis of images from cells stained with Propidium iodide (PI). The apoptotic effect was evaluated by Annexin V/7-Amine-Actinomycin D (AAD) staining and caspase-3/7. Antiproliferative activity was determined by Carboxyfluorescein succinimidyl ester (CFSE) staining. The extract, fractions, and aqueous residue (AR) exhibited cytotoxic activity against the PANC-1 cell line, with EAF showing the highest activity, demonstrating an IC_50_ of 26.27 µg/mL. In contrast, EAF showed lower cytotoxicity against HaCaT cells. Likewise, EAF inhibited proliferation and induced cell death via apoptosis in a concentration-dependent manner. β-sitosterol, palmitic acid, and apigenin-8-*C*-glucoside were isolated from EAF, whereas quercetin and gallic acid were identified in a mixture obtained from this fraction. Therefore, this species could be considered as an important source for cancer therapy.

## 1. Introduction

Cancer is one of the leading causes of death worldwide. It is a heterogeneous and multifactorial disease in which a series of genomic/molecular alterations cause uncontrolled cell growth and proliferation [1]. Cancer cells acquire various molecular mechanisms to maintain proliferative signaling and resistance to cell death [2]. Recent evidence indicates the fundamental role of apoptosis in maintaining tissue homeostasis, contributing to the elimination of unnecessary and unwanted cells to maintain a balance between cell survival and death. Therefore, the induction of apoptosis in tumor cells constitutes a specific therapeutic approach for cancer chemotherapy [3]. There are currently conventional therapies available to treat cancer, such as radiation therapy, immunotherapy, and surgery, with chemotherapy being the most used first-line treatment. However, anticancer agents often have limitations such as unwanted side effects, multiple resistance mechanisms, limited therapeutic efficacy, and high costs [4], indicating a need to develop new approaches to cancer therapy with the aim of promoting cancer cell death without causing extensive damage to normal cells. Among the various types of cancer, pancreatic cancer poses a significant clinical challenge due to its poor prognosis and limited therapeutic options, particularly in advanced stages. According to GLOBOCAN estimates for 2024, nearly 532,000 new cases and approximately 491,000 deaths from this disease were recorded worldwide, making it the sixth-leading cause of cancer-related death. These figures underscore the importance of investigating compounds that demonstrate activity against cancer cell models and contribute to the identification of new candidate molecules for preclinical studies [5]. In this regard, natural products derived mainly from plant species have proven to be a potentially diverse source of chemical compounds used in cancer treatment [6].

Species of the genus *Euphorbia* constitute a significant source of secondary metabolites characterized by structural diversity and pharmacological potential [7]. Their use in traditional medicine has been linked to the presence of various bioactive compounds, notably triterpenes based on skeletons such as cycloartane, lupane, oleanane, ursane, and taraxane, as well as diterpenes, including those of the jatrophane, lathyrane, ingenane, and tigliane types. Additionally, phenolic compounds and flavonoids have been identified in various species of this genus [8,9]. Numerous in vitro studies have documented the cytotoxic and antiproliferative activities of specific *Euphorbia* metabolites and extracts, sparking interest in their potential effects on cancer cell viability and apoptosis-related mechanisms. Collectively, these findings support phytochemical and pharmacological research on species of this genus as a strategy for identifying bioactive metabolites with potential application in the development of new therapeutic agents [10,11].

*Euphorbia indivisa* belongs to the *Euphorbia* genus and is one of the least studied species; little is known regarding its biological activities. This plant belongs to a group of weeds common in Mexico and is popularly known as golondrina, alfombrilla, or hierba de la golondrina. Generally, this species is short-lived and grows in a prostate manner, with branches that can measure up to 30 cm long and abundant milky liquid that flows from any wound or incision [12]. *E. indivisa* has different scientific synonyms such as *Chamaesyce inaequalis*, *Chamaesyce indivisa*, *Euphorbia adenoptera*, *Euphorbia dioeca*, and *Euphorbia multifoliosa* (https://wfoplantlist.org). The aim of this research was to evaluate the cytotoxic activity of the extract and fractions obtained from the aerial parts of *E. indivisa*, as well as to evaluate the cell death by apoptosis induced by the sample with the highest activity and to identify the major secondary metabolites responsible for this therapeutic effect.

## 2. Results

### 2.1. Screening of Cytotoxic Effects of E. indivisa on Different Cancer Cell Lines

**Figure 1:**
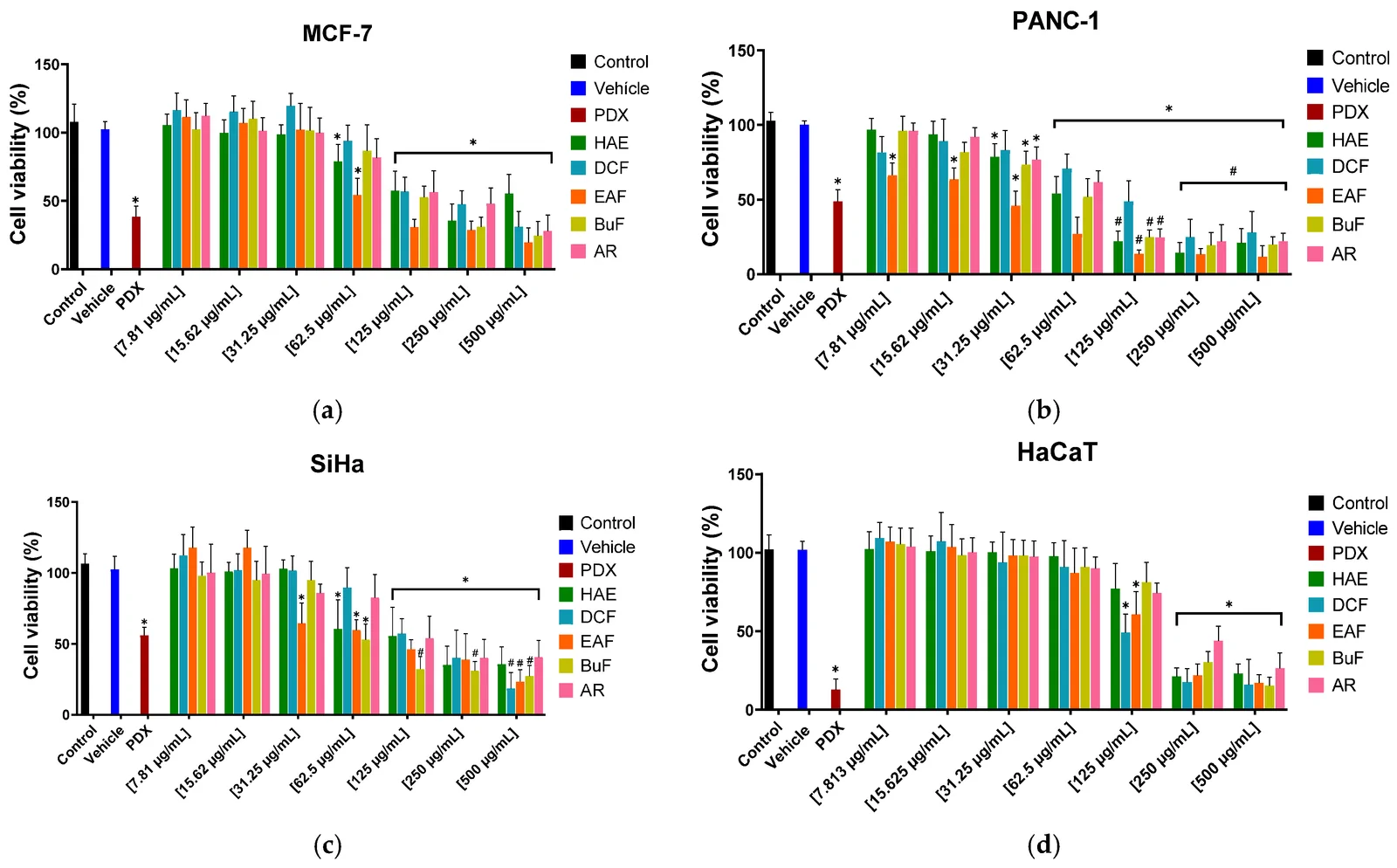
*Euphorbia indivisa* extract and fractions decrease cell viability in cancer and nontumorigenic cell lines. Cell viability of the hydroalcoholic extract (HAE); the dichloromethane (DCF), ethyl acetate (EAF), and butanol (BuF) fractions; and the aqueous residue (AR) of *Euphorbia indivisa* against MCF-7 (**a**), PANC-1 (**b**), SiHa (**c**), and HaCaT (**d**) cell lines at concentrations ranging from 7.81 to 500 µg/mL for 48 h. Dimethyl sulfoxide (DMSO) was used as a vehicle control and podophyllotoxin (PDX) at 12.5 µg/mL as a positive control. Data represent the mean ± standard deviation (SD) of more than 3 independent experiments. *: different from vehicle, *p* < 0.05; ^#^: different from positive control, *p* < 0.05.

The inhibitory effect of the HAE and its fractions on cell viability was evaluated in different cell lines over a 48 h period. Figure 1 shows that exposure to the different samples altered the viability of the cell lines evaluated, with responses varying depending on the sample, the concentration, and the cell line. In the PANC-1 cell line, treatment with concentrations of 62.5 µg/mL and above of all the treatments was able to effectively inhibit cell viability compared with the MCF-7 and SiHa cell lines; differences in all treatments ere evident starting at 125 µg/mL.

The HAE and all fractions (DCF, EAF, BuF, and AR) showed higher cytotoxic activity and exhibit lower IC_50_ values against the PANC-1 cell line compared with the other cell lines (Table S1). Among the samples evaluated, the EAF fraction exhibited the highest cytotoxic activity, with an IC_50_ value of 26.27 µg/mL against PANC-1 cells. However, EAF did not exhibit the same cytotoxic activity against the MCF-7 (117.2 µg/mL) and SiHa (126.2 µg/mL) cell lines, as the IC_50_ values were higher in these cell lines.

Additionally, the nontumor HaCaT cell line was less susceptible to the cytotoxicity of EAF, presenting an IC_50_ value of 155.2 µg/mL, suggesting a differential effect between nontumor and tumor cells. The selectivity indexes (SI) of HAE and the fractions were calculated as previously described. In this analysis, no differential cytotoxicity (SI < 3) was observed for the extract or fractions against the MCF-7 and SiHa lines compared with HaCaT cells (Table S2). Only the HAE, EAF, BuF, and AR samples exhibited selectivity indices (SI) greater than 3 against the PANC-1 cell line. Among these, EAF showed the highest SI, suggesting a greater cytotoxic effect on PANC-1 cells compared to HaCaT cells under the experimental conditions evaluated. Based on this result, EAF was selected for further studies.

### 2.2. Analysis of Cell Confluence and Cell Death Using the IncuCyte SX1 System

**Figure 2:**
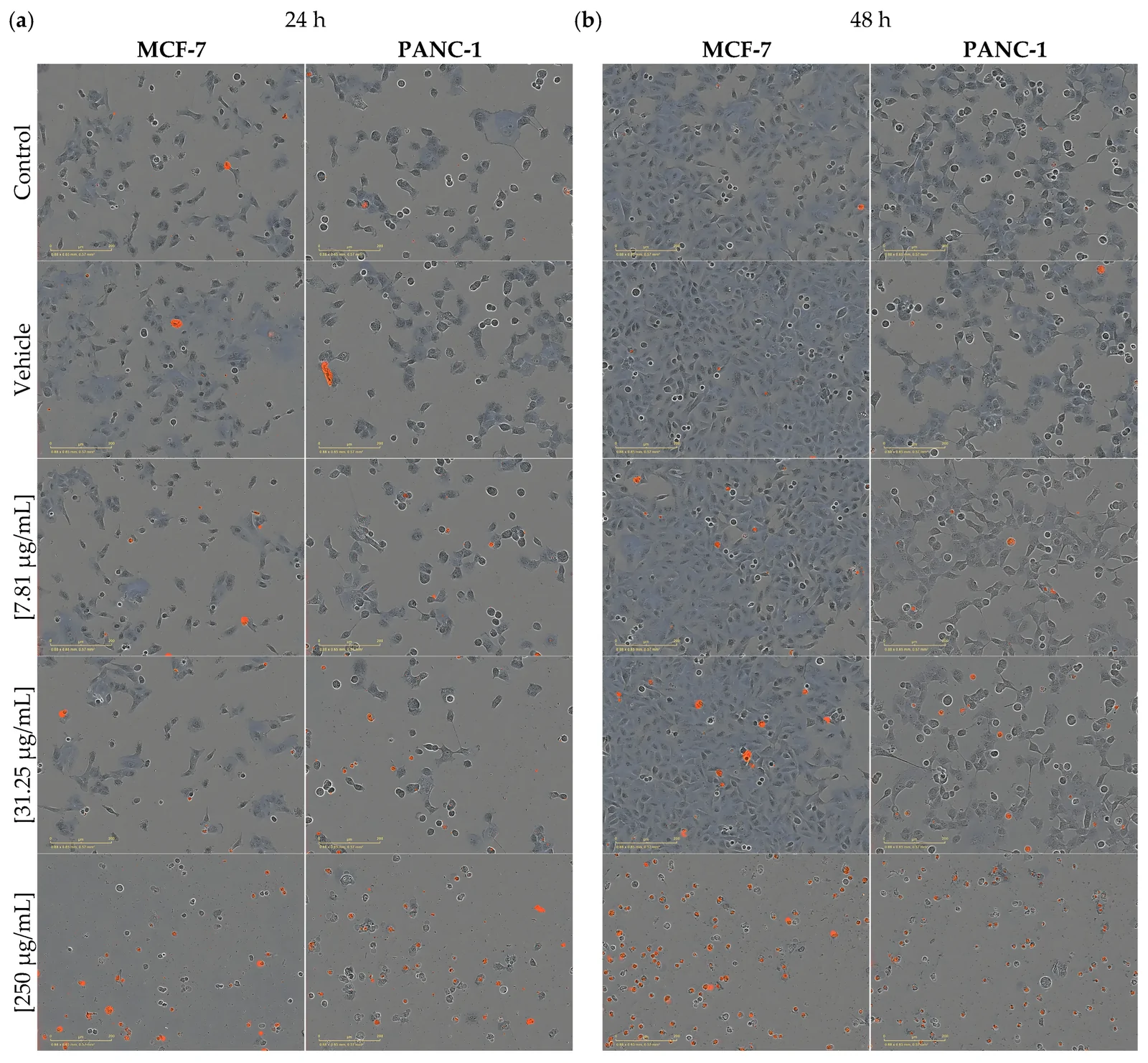
EAF treatment induces cell death in MCF7 and PANC-1 cells. Overlayed phase–contrast and fluorescence images (20× magnification) of MCF-7 and PANC-1 cells after 24 h (**a**) and 48 h (**b**) of treatment with DMSO (vehicle) and the indicated concentrations of EAF, in the presence of PI. Representative images of cells treated with the lowest, intermediate, and one of the highest concentrations evaluated are shown, obtained from more than three independent experiments. Scale bar represents 200 µm. 0–200 µm: corresponds to the image scale, represented by a scale bar. 0.88 × 0.65 mm: corresponds to the dimensions of the field of view (width × height). 0.57 mm^2^: corre-sponds to the approximate area of that field of view.

Based on the results obtained from IC_50_, the effect of EAF on cell confluence was evaluated as an indicator of the density and growth status of the culture in two cancer cell lines (MCF-7 and PANC-1), while cell death was evaluated using the dye propidium iodide (Figure 2).

**Figure 3:**
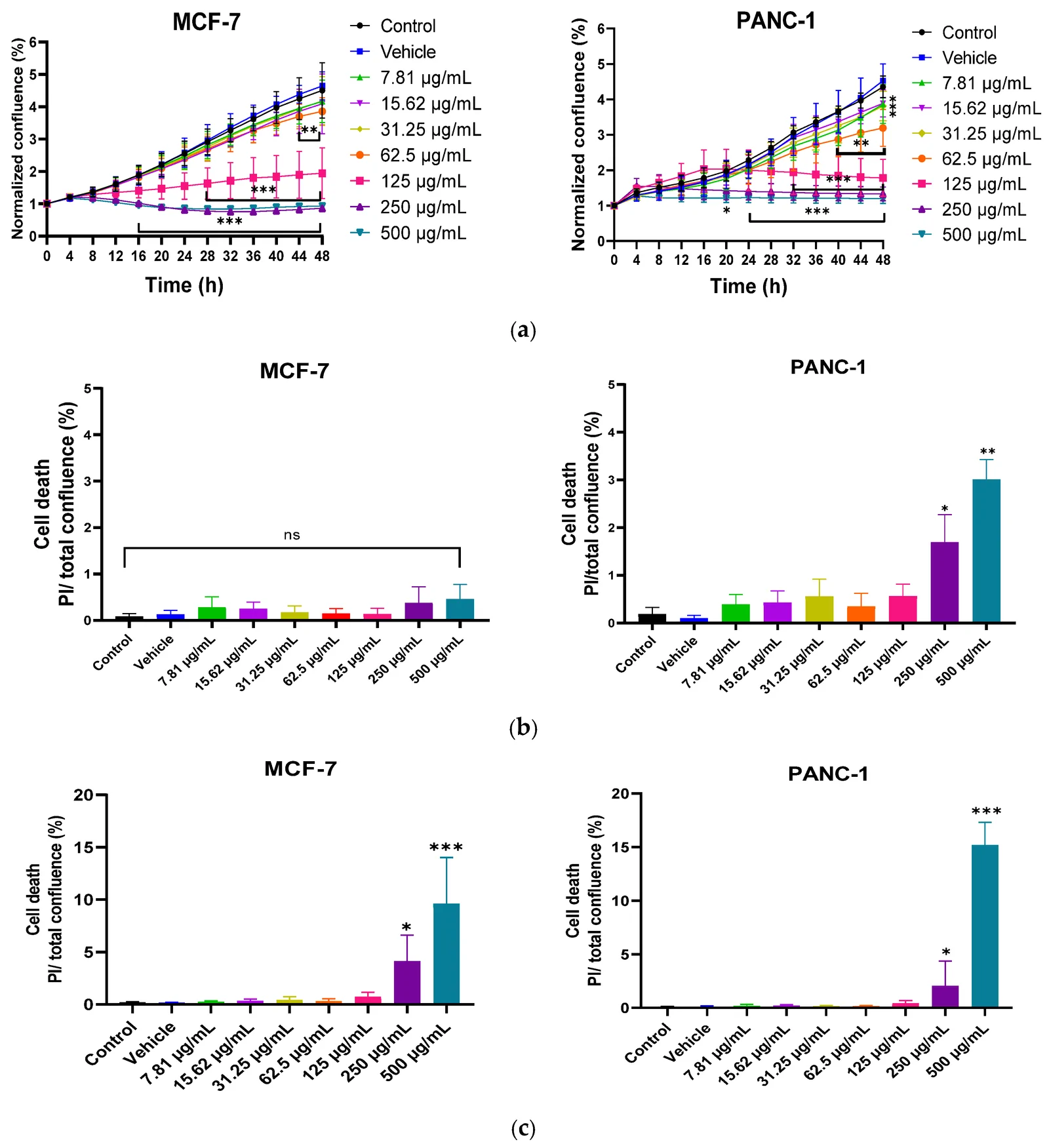
EAF treatment decreases cancer cell proliferation and induces cell death. Cells were treated with EAF at the indicated concentrations. Cell proliferation was evaluated as changes in cell confluency by the IncuCyte SX1 real-time cell imaging system (**a**). Cell death was evaluated as PI staining [% confluency of PI (+) cells] and normalized to total % confluency at 24 h (**b**) and at 48 h (**c**). Graphs show the mean ± SD of more than 3 independent experiments. *: different from vehicle, *p* < 0.05; **: different from vehicle, *p* < 0.01; and ***: different from vehicle, *p* < 0.001. ns (not significant) *p* > 0.05 compared with vehicle.

Real-time automated phase contrast and red fluorescence images were acquired using the IncuCyte SX1 system at different concentrations and time points. Cell confluence curves were generated from these images, and quantitative data on cell death were obtained (Figure 3). The data obtained show that the treatments significantly decreased the cell confluence in both cancer lines compared with the vehicle, indicating a reduction in confluence dependent on concentration and exposure time.

The PANC-1 cell line showed sensitivity to treatment with different concentrations of EAF starting at 24 h, as the higher concentrations induced an increase in propidium iodide (PI) staining, indicating an increase in cell death. Likewise, at 48 h, the lowest concentrations reduced cell confluence, suggesting an effect on cell growth (Figure 3a). In contrast, the MCF-7 cell line did not show a significant increase in cell death 24 h after treatment. However, higher concentrations of EAF reduced cell confluency after 48 h of exposure (Figure 3a,c). These results suggest that higher concentrations of EAF induce cell death in both lines, while lower concentrations cause a decrease in cell confluence at an exposure time of 48 h. However, this decrease in confluence was not considered direct evidence of cell death, as it could also be associated with the inhibition of proliferation, changes in cell morphology, or cell detachment. Therefore, cell death was assessed using propidium iodide (PI) staining a marker that identifies cells with a loss of plasma membrane integrity. As shown in Figure 3b,c, higher concentrations led to an increase in the PI signal, indicating a greater proportion of cells with compromised plasma membrane integrity and supporting the existence of a cytotoxic effect under these experimental conditions.

### 2.3. Activation of Caspase-3/7 Induced by EAF in MCF-7 and PANC-1 Cell Lines

**Figure 4:**
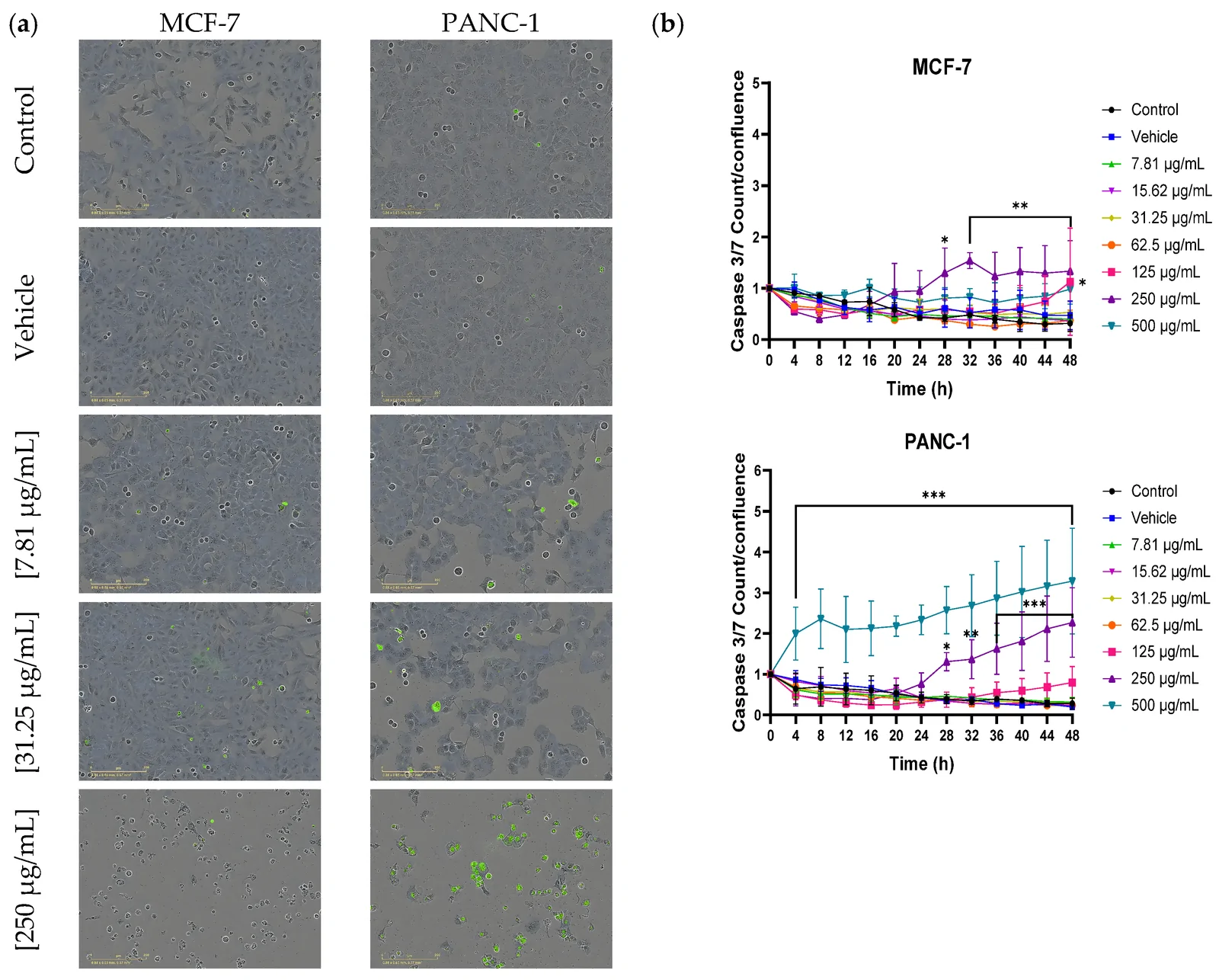
EAF induces apoptosis in MCF-7 and PANC-1 cancer cell lines. IncuCyte SX1 analysis of caspase-3/7 activity with EAF over time. (**a**) Representative images of caspase-3/7 induction at 48 h. Images were taken with a 20× objective lens. (**b**) Graphic representation of the amount of caspase-3/7 induction per well with DMSO and EAF (7.81–500 µg/mL) over time. Results are presented as mean ± SD; all samples were measured independently in triplicate. *: different from vehicle, *p* < 0.05; **: different from vehicle, *p* < 0.01; ***: different from vehicle, *p* < 0.001. 0–200 µm: corresponds to the image scale, represented by a scale bar. 0.88 × 0.65 mm: corresponds to the dimensions of the field of view (width × height). 0.57 mm^2^: corresponds to the approximate area of that field of view.

The assessment of combined caspase-3/7 activity was performed directly using the IncuCyte SX1 imaging system (Figure 4a). Functional caspase activity was kinetically monitored as a measure of the induction of apoptosis after treatment in MCF-7 and PANC-1 cell lines with different concentrations of EAF. After 48 h of treatment, caspase-3/7 was demonstrated to be activated in both cell lines compared with untreated cells. Only the highest concentrations of EAF (250 and 500 µg/mL) showed pronounced increases in activated caspase-3/7 at 24 h of treatment, as well as a faster induction of caspase activity in the PANC-1 cell line compared with the MCF-7 cell line (Figure 4b). Consistent with previously obtained results, higher concentrations of EAF increased caspase-3/7 activity and propidium iodide (PI) staining, suggesting that the treatment induces a cell death process consistent with apoptosis.

### 2.4. Apoptosis Induced by EAF in PANC-1 Cells

**Figure 5:**
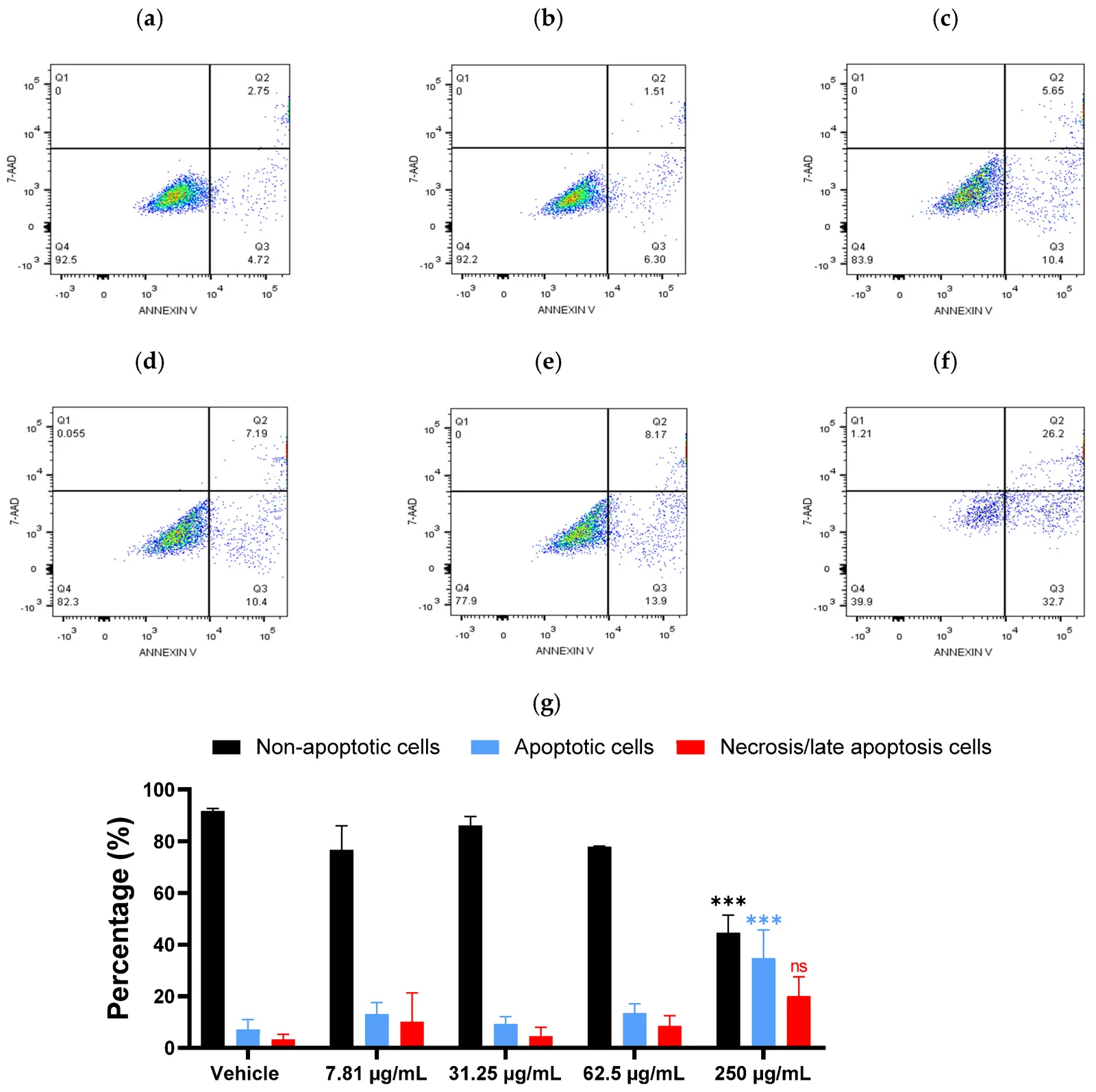
EAF induces apoptosis in PANC-1 cells. The apoptosis of PANC-1 cells was detected by Annexin V/7-AAD staining and flow cytometry. (**a**–**f**) Apoptosis stage diagram of PANC-1 cells for 24 h treatment with different concentrations of EAF. Control (**a**), vehicle (**b**), 7.81 µg/mL (**c**), 31.25 µg/mL (**d**), 62.5 µg/mL (**e**), and 250 µg/mL (**f**). Cells in quadrants Q1/Q2, Q3, and Q4 correspond to necrosis/late apoptosis cells, apoptotic cells, and nonapoptotic cells, respectively (**g**). Data represent the mean ± SD (n = 3). Significant differences are indicated as follows: *** indicates *p* < 0.001. ns indicates not significant.

With the aim of confirming the induction of apoptosis by detecting the externalization of phosphatidylserine, the Annexin V assay was performed on the cell line that showed the highest activation of caspase-3/7. The results in Figure 5a–f show that at different concentrations of EAF, the proportion of cells decreases significantly. At a concentration of 250 µg/mL, the proportion of nonapoptotic cells decreased to 40%, cells in early apoptosis increased to around 33%, and the remaining 28% of cells were those in late apoptosis/necrosis.

Figure 5g shows the average number of events present in each quadrant from the replicates performed. It can be seen that there is no significant difference between the three lower concentrations of EAF compared with the untreated group; only the highest concentration showed a significant difference in apoptotic cells compared with the vehicle, with an increase in the percentage of cells dying by apoptosis after treatment with 250 µg/mL of EAF for 24 h. This difference, compared with the control group, was significant.

Taken together, the activation of caspase-3/7 and the increase in PI staining suggest that the treatment induces a cell death process consistent with apoptosis. Furthermore, the concentration-dependent response observed in both assays supports the potential of *E. indivisa* to induce apoptosis.

### 2.5. EAF Inhibits Cell Proliferation in the PANC-1 Line

A flow cytometry analysis using CFSE was performed on PANC-1 cells to assess cell proliferation by tracking the dilution of fluorescence, which progressively decreases with each cell division.

**Figure 6:**
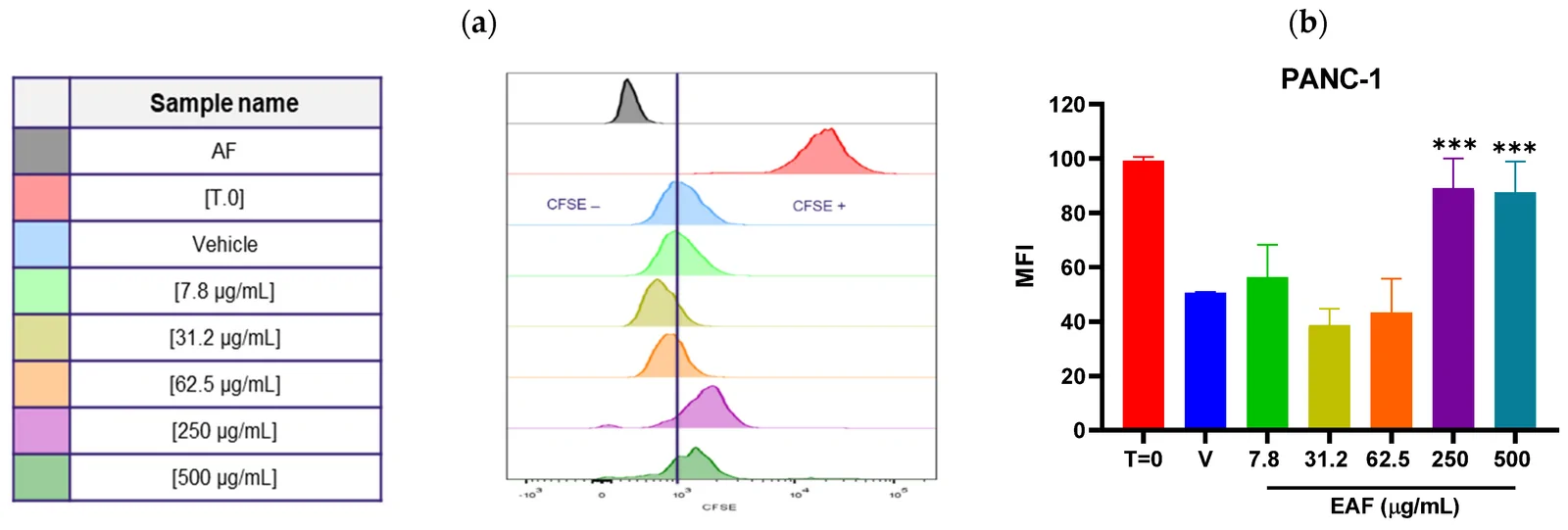
EAF of *E. indivisa* inhibits the proliferation of pancreatic cancer cells. CFSE− stained PANC-1 cells were analyzed by flow cytometry after 48 h of treatment at different concentrations (7.8, 31.2, 62.5, 250, and 500 µg/mL). DMSO was used as the vehicle, and the autofluorescence (AF) of unlabeled cells and time zero (T.0) of stained cells (**a**) were used as controls. Graph of mean fluorescence intensity (MFI) (**b**). Data represent the mean ± SD (n = 3). Significant differences are indicated as *** *p* < 0.001.

The CFSE histogram represents the distribution of PANC-1 cells with respect to CFSE fluorescence levels. Cells with fewer divisions showed higher CFSE intensity, observed on the right side of the histogram (CFSE+), while cells with more divisions showed lower CFSE intensity, observed on the left side (CFSE−), as is the case with the controls (Figure 6a). The cells were affected by treatment with EAF after 48 h in contact with the samples. EAF showed a more significant inhibition of cell proliferation at higher concentrations (250 and 500 µg/mL) compared with treatment with other concentrations, which showed no significant difference compared with the vehicle (Figure 6b).

The decrease in cell proliferation observed after treatment correlates with the results obtained in cell death induction, as evidenced by apoptosis assays, which limits the number of cells capable of completing cell division cycles. However, the activity observed at high concentrations can be attributed to the complex nature of the fraction that positions EAF as a source in the search for bioactive metabolites that can be used in the investigation of new anticancer drugs to combat pancreatic cancer.

### 2.6. Phytochemical Study

The results of biological assays demonstrated the pharmacological importance of the secondary metabolites present in the species *E. indivisa*. Of the samples evaluated, EAF showed the greatest activity in the cytotoxic evaluation. The compounds present in this fraction were identified and included the following: β-sitosterol (1), palmitic acid (2), and apigenin-8-*C*-glucoside (5) were isolated individually, whereas quercetin (3) and gallic acid (4) were identified in a mixture.

Furthermore, compounds such as isomultiflorenol (6) and taraxasterol (7) were also identified in DCF (Figure S1). The structures of the molecules were identified by analysis of their spectroscopic data obtained from 1D NMR and corroborated by comparison with data reported in the literature [13,14,15,16,17,18,19,20,21]. Due to the complexity of DCF, further compounds were identified by comparing their obtained mass spectra with those reported in the NIST14 spectral library and by comparing the retention indices reported in the literature. Analysis of *E. indivisa* by GC-MS revealed the presence of phytol (8), α-tocospiro A (9), 13,27-cycloursan-3-ol (10), α-amyrin (11), β-amyrin (12), lanosterol (13), stigmast-4-en-3-ona (14), and lupeol (15) (Table S3) [22,23,24,25,26,27,28,29,30,31,32]. Finally, the compound identified in the polar n-butanol fraction (BuF) was shikimic acid (16). According to the reviewed literature, most of the compounds identified in the aerial parts of *E. indivisa* have already been reported in other species of the same genus. The phytochemical profile obtained for *E. indivisa* is consistent with the broad chemical diversity previously documented in the genus *Euphorbia* [33,34,35,36,37,38]. Previous studies have reported a wide variety of flavonoids and other phenolic compounds, as well as terpenoids, which are dominant constituents of these species, making them a rich source of therapeutic natural products with diverse pharmacological activities (Table 1).

## 3. Discussion

The use of medicinal plants as a strategy for cancer prevention and treatment has been researched for many years, as this disease is considered one of the leading causes of death worldwide [46]. Numerous studies have reported the cytotoxic potential of various plant species of the genus *Euphorbia* against cancer cell lines, with the aim of exploring new sources of anticancer agents, due to the presence of various groups of secondary metabolites responsible for these activities [47].

In the present study, we investigated the cytotoxic effects of *E. indivisa* on three human cancer cell lines, including breast, pancreas, and cervical cancer. We observed that EAF exhibited cytotoxic effects on human cancer cell lines tested, with the highest effect (reduced viability) for the pancreas cancer line. According to the American National Cancer Institute criteria, EAF is a promising source of compounds for further analysis, since its IC_50_ values were lower than 30 µg/mL (http://www.cancer.gov). EAF exhibited cytotoxic activity against PANC-1 cells, with an IC_50_ of 26.27 µg/mL. Compared with HaCaT cells, these results indicate greater sensitivity of PANC-1 cells to the fraction under the experimental conditions evaluated. These results indicate that the fraction exerts a more pronounced cytotoxic effect on pancreatic cancer cells than on non-cancerous cells, suggesting a differential effect between nontumor and tumor cells. For comparison, a previous study reported by Jaros et al., evaluating a silver diclofenac coordination polymer in pancreatic cancer models, reported IC_50_ values of 12.5 µM and 26.43 µM for cisplatin in PANC-1 and HaCaT cells, respectively, corresponding to an SI of approximately 2.11 [48]. Although the SI obtained for EAF was numerically higher, direct comparisons between studies should be interpreted cautiously because differences in experimental conditions and assay procedures may influence the results. Therefore, the SI of 5.90 should be interpreted as an indication of differential sensitivity between the two cell models evaluated. However, because HaCaT cells are immortalized human keratinocytes and do not represent a non-malignant pancreatic counterpart of PANC-1, this result provides an initial indication of differential sensitivity that warrants further evaluation using tissue-matched non-malignant pancreatic cells.

Our results are consistent with reports of moderate cytotoxic activity in other species of the genus *Euphorbia*. For example, Youssef et al. evaluated the cytotoxic activity of an extract from *Euphorbia paralias* against five tumor cell lines (U87, MCF-7, A549, PANC-1, and DLD-1) and observed the highest activity against PANC-1, with an IC_50_ value of 60.11 ± 26.1 µg/mL a value comparable to that obtained in the present study for the EAF of *E. indivisa*. For the MCF-7 cell line, the authors reported an IC_50_ of 74.6 ± 15.6 µg/mL, which is lower than the value determined for *E. indivisa* (117.2 µg/mL), indicating a weaker cytotoxic effect for the sample evaluated in our study against this cell line. These differences may be related to variations in the chemical composition and the abundance of secondary metabolites present in both species. Such factors could contribute to the observed variations in cytotoxic activity against different tumor cell lines.

To gain more insight into the role played by EAF in cancer, we investigated the biological effect of EAF over the cell confluence and the ability of MCF-7 and PANC-1 to induce cell death. The results indicated that the ability of fractions to reduce the percentage of cell confluence is dependent on concentration and exposure time. The data obtained were consistent with the results determined by the SRB method. According to the data, kinetic curves of cell confluence were generated as a function of time, showing a decrease in cell growth rate throughout the treatment period compared with the control. A reduction in cell confluence was observed at certain concentrations, although this change was not always accompanied by an increase in the PI-associated signal. These findings suggest that EAF can affect cell growth or proliferation; however, confluence measurements alone do not allow one to distinguish between an antiproliferative effect and a cytotoxic one. Consequently, these measurements provide complementary information regarding cell growth and PI-associated events, which may be linked to concentration-dependent antiproliferative or cytostatic effects or to other changes in cell morphology and adhesion. Furthermore, this behavior indicates that the evaluated fraction not only affects cell viability but could also interfere with cell cycle processes or alter cell adhesion mechanisms [49].

For the evaluation of cell death, the results displayed a significant increase in the PI+ cell population after treatment with the samples, indicating a loss of plasma membrane integrity and, consequently, a decrease in cell viability. This effect was time-dependent for the MCF-7 line and concentration-dependent for both cell lines (MCF-7 and PANC-1). Taken together, the results of the confluence and cell death assays indicate that the treatment may initially exert a cell growth inhibition effect or cytostatic activity, followed by the activation of cell death mechanisms at higher concentrations.

Similarly, the assessment of caspase-3/7 activity revealed that only the highest concentrations of EAF significantly increased their activity in PANC-1 and MCF-7 cell lines. Morphological changes in the cells were also observed compared with the vehicle, suggesting the involvement of a cell death process consistent with apoptosis. It is well documented that MCF-7 cells lack functional caspase-3 due to a deletion in the *CASP3* gene. Consequently, the fluorescence signal observed in our assays is attributed primarily to the activation of caspase-7 rather than caspase-3. In PANC-1, the activation of caspase-3/7 was earlier and more pronounced than in MCF-7, indicating greater sensitivity of these cells to EAF treatment. This differential response may be related to the unique biological characteristics of each cell line, which influence susceptibility to the activation of treatment-induced cell death pathways. However, further studies such as caspase inhibition assays will be necessary to confirm whether the observed cell death specifically depends on the activation of these proteases.

Based on the results observed in the PANC-1 cell line, this line was selected for further characterization of the type of cell death using the Annexin V/7-AAD assay. The results showed that higher concentrations of EAF significantly increased the percentage of Annexin V-positive cells, indicating exposure of phosphatidylserine on the cell surface—one of the characteristic early events of apoptosis. In contrast, although an increase in the population of 7-AAD-positive cells was observed at the highest concentrations of EAF, this increase was not statistically significant compared with the vehicle. The combination of Annexin V with the dye 7-amino-actinomycin (7-AAD) allows the identification of different states of the plasma membrane during the cell death process, distinguishing apoptotic cells from dead (late apoptotic or necrotic) cells, since 7-AAD is a nucleic acid intercalator that penetrates the plasma membrane of dead cells [50]. These findings suggest that under the experimental conditions evaluated, treatment with EAF predominantly induces early events of apoptosis in PANC-1 cells rather than a generalized loss of plasma membrane integrity associated with late stages of cell death.

This study also evaluated the antiproliferative activity of EAF from *E. indivisa*. CFSE analysis showed a reduction in the proliferative capacity of PANC-1 cells following treatment with EAF, particularly at higher concentrations. However, these concentrations were also associated with increased cell death, as evidenced by the PI and apoptosis assays. Therefore, the reduction in proliferation observed at higher EAF concentrations cannot be attributed exclusively to a direct cytostatic effect and may be partly secondary to the loss of viable cells, representing a combination of cytostatic and cytotoxic effects of the fraction.

The biological activity observed in EAF may be related to the presence of various secondary metabolites that, collectively, may contribute to the observed cytotoxic effect. In this fraction, compounds such as β-sitosterol, palmitic acid, quercetin, gallic acid, and apigenin-8-*C*-glucoside were identified. These compounds have previously been reported to exhibit cytotoxic, antiproliferative, and apoptotic activities in various cell lines [51,52,53,54,55,56,57,58,59]. Therefore, the activity observed in EAF may be attributed to the presence of these compounds. However, because the individual compounds were not evaluated, it is not possible to determine which of them contributes most significantly to the observed activity. Quantitative compositional analysis, followed by the evaluation of isolated compounds and appropriately designed combination studies, will be necessary to clarify the relationship between the chemical composition of EAF and its biological activity.

Meanwhile, various metabolites were identified in the other fractions, including phytol, 13,27-cycloursan-3-ol, α-amirine, β-amirine, lanosterol, lupeol, α-tocospiro A, γ-sitosterol, taraxasterol, isomultiflorenol, and shikimic acid. Some of these have been reported to have cytotoxic, apoptotic, antioxidant, and anti-inflammatory effects, among others [60,61,62,63]. However, despite the diversity of metabolites identified in these fractions, their cytotoxic activity was lower than that observed for EAF. This difference could be related to variations in the chemical composition or concentration of the compounds, as well as to possible interactions between them. One limitation of this study is that cytotoxic and apoptotic activities were evaluated in EAF; therefore, it is not possible to attribute the biological effect to an individual compound or confirm synergistic mechanisms, aspects that will need to be addressed in future studies with pure compounds.

In this context, the present study is the first report demonstrating the potential of EAF from *E. indivisa* to induce a cytotoxic response consistent with apoptosis and to reduce proliferative capacity in a pancreatic cancer cell line. It is interesting to note that pancreatic cancer is one of the most aggressive and lethal types of cancer. It has a low survival rate and is difficult to detect early [64], which positions the *E. indivisa* species as a source of potential anticancer secondary metabolites to treat one of the most lethal types of cancer. The identification of quercetin, gallic acid, apigenin-8-*C*-glucoside, β-sitosterol, and palmitic acid, along with other terpene and sterol constituents, expands the available phytochemical information on *E. indivisa*. Although these metabolites are not necessarily unique to this species, documenting their presence contributes to characterizing its chemical composition and correlating it with the biological properties that have been evaluated.

## 4. Materials and Methods

### 4.1. Chemicals and Reagents

Industrial-grade solvents and other reagents were acquired from local distributors. Deuterated solvents chloroform (CDCl_3_), methanol (CD_3_OD), and dimethyl sulfoxide (DMSO-*d*_6_) were used for the NMR experiments, as well as silica gel 60 for column chromatography (CC) (70–230 mesh), silica gel 60 for flash column chromatography (230–400 mesh), and aluminum sheets 20 × 20 cm kieselgel 60 F_254_ for thin-layer chromatography. Furthermore, sulforhodamine B (SRB), dimethyl sulfoxide (DMSO), propidium iodide (PI), podophyllotoxin (PDX), penicillin, streptomycin, and trypsin were purchased from Sigma-Aldrich (Toluca, Mexico). RPMI-1640, DMEM, and fetal bovine serum (FBS) culture media were purchased from Biowest (Mexico City, Mexico). The Annexin V Apoptosis Detection Kit with 7-AAD Kit was purchased from BioLegend (San Diego, CA, USA). IncuCyte Caspase-3/7 Green Apoptosis Assay Reagent was purchased from Essen Bioscience (Sartorius AG, Göttingen, Germany). The CFSE CellTrace™ Cell Proliferation Kit were purchased from Invitrogen/Thermo Fisher Scientific (Eugene, OR, USA).

### 4.2. Plant Material

The aerial parts of *Euphorbia indivisa* were collected in Tepic Nayarit (21°28′56.0″ N 104°50′52.4″ W) in October 2022. The plant was identified by Dr. Ana María Hanan Alipí, and a voucher specimen (voucher specimen number: 1753) was deposited in the Universidad Autónoma de Nayarit Herbarium.

### 4.3. Extraction and Isolation

Aerials parts of *E. indivisa* were dried at room temperature and subsequently ground into fine powder. Then, 1.067 kg of the material was macerated in a bottle with 10 L of a solution of 85% methanol for three days at room temperature. Afterward, the solvent was filtered and evaporated to dryness using a rotavapor with reduced pressure. This last process was performed in triplicate, and all residues were pooled to obtain 77.61 g of hydroalcoholic extract (HAE). Dichloromethane (DCF), ethyl acetate (EAF), and butanol (BuF) fractions, as well as aqueous residue (AR), were obtained through liquid–liquid extractions from the HEA. The compounds were isolated using column chromatography, employing silica gel (70–230 and 230–400 mesh) as the stationary phase. Elution was carried out using CH_2_Cl_2_, followed by a step gradient of CH_2_Cl_2_: acetone in the following proportions: [100:0; 90:10; 80:20; 70:30; 60:40; 50:50; 0:100]. Fractions of 125 mL were collected and monitored by thin-layer chromatography (TLC), using F_254_ silica gel plates as the stationary phase. *n*-hexane, CH_2_Cl_2_, EtOAc, methanol, and acetone were used as mobile phases in different gradients. The TLC plates were visualized under ultraviolet light at 254 and 366 nm wavelengths and then developed via spraying with a solution of (NH_4_)_4_Ce(SO_4_)_4_ in 2 N H_2_SO_4_.

### 4.4. GC-MS and NMR Analyses

The compounds for which purification was not achieved by column chromatography (CC) were identified by gas chromatography–mass spectrometry (GC-MS) using electron impact ionization at 70 eV on a 7890B gas chromatograph (GC) and 7000D triple quadrupole mass spectrometer (QQQ) from Agilent Technologies, with a HP-5MS column (30 m × 0.25 mm × 0.25 µm) and helium as the carrier gas at a flow rate of 1 mL/min. The compounds were identified by comparing the obtained mass spectra with those in the NIST14 spectral library (National Institute of Standards and Technology) using Agilent Version 14 software. The compound assignments were also corroborated by comparing the retention indices reported in the literature. On the other hand, Bruker AVANCE III HD 500 MHz (11.74T) and Jeol ECZ 600 MHz (14.1T) spectrometers were used to carry out ^1^H and ^13^C experiments to determine the structures of the isolated compounds. The results obtained were analyzed and compared with data reported in the literature for compounds that have been previously identified.

### 4.5. Cytotoxic Activity

#### 4.5.1. Cell Lines and Culture Conditions

The assays were performed on three human cancer cell lines: breast cancer (MCF-7; ATTC No. HTB-22), cervical cancer (SiHa; ATTC No. HTB-35), and pancreatic cancer cell lines (PANC-1; ATTC No. CRL-1469). To perform the selectivity index, tests were assayed on immortalized human keratinocyte cell lines (HaCaT; human skin keratinocytes). PANC-1 and HaCaT cell lines were donated by Dr. Enrique Pérez Cardenas of the National Cancer Institute of Mexico. Cancer cell lines were cultured in RPMI-1640 medium, while nontumorigenic HaCaT cells were cultured in DMEM; both media were supplemented with 10% SFB and 1% antibiotic of penicillin–streptomycin. All cell lines were incubated at 37 °C in an atmosphere with 5% CO_2_ and 100% humidity.

#### 4.5.2. Cell Viability Measurement

The SRB (cat. 230162, Sigma-Aldrich) assay was used for cell density determination based on the measurement of cellular protein content and according to the methodology described by Vichai & Kirtikara [65]. The method described here has been optimized for screening the cytotoxic activity of *E. indivisa* extract and fractions. After reaching 80% confluence in the plates, the cells were treated with the extract and fractions at different concentrations (7.81–500 µg/mL) for 48 h. DMSO was used as a vehicle and PDX as a positive control. After an incubation period, cell monolayers were fixed in 30% trichloroacetic acid for 1 h at 4 °C and stained with SRB for 30 min. Subsequently, the excess dye was removed by repeated washing with 1% acetic acid. The protein-bound dye was dissolved in 10 mM of Tris base solution, and the optical density (OD) at 510 nm was read in a microplate reader. The survival percentage of treated cells over the control cells ×100 (T/C) was calculated as cell viability:

% cell viability = (OD (test sample) − OD (blank))/(OD (control) − OD (blank)) × 100

IC_50_ values were determined by nonlinear regression analysis of the concentration–response curves using GraphPad Prism. Treatment concentrations were expressed on a logarithmic scale, and the response was normalized between 0 and 100%. The “log(inhibitor) vs. normalized response” model was used for curve fitting, which assumes a sigmoidal concentration–response relationship. IC_50_ values were calculated from the LogIC_50_ parameter obtained from the nonlinear regression. The goodness of fit was evaluated using the coefficient of determination (R^2^), and 95% confidence intervals (CIs) for the IC_50_ estimates were calculated using the profile likelihood method.

#### 4.5.3. Selectivity Index (SI)

The selectivity index was obtained by dividing the half-maximum inhibitory concentration (IC_50_) for a nontumorigenic cell (HaCaT) by the IC_50_ for a tumor cell. SI values less than 3 indicate a lack of selectivity toward cancer cells [66]. The SI values of the extract and fractions of *E. indivisa* were determined using the following equation:

*SI* = (IC_50_ for nontumorigenic cell)/(IC_50_ for a tumor cell)

### 4.6. Confluence and Cell Death Assays

Cell confluence was calculated using a live-cell IncuCyte SX1 System (Sartorius, Göttingen, Germany). The cells of MCF-7 and PANC-1 were seeded in 96-well plates with 4 × 10^3^ cells/well in 100 µL of medium. Afterward, the cells were placed in contact with the controls and the different concentrations of EAF (7.81–500 µg/mL) for 48 h. Four images per well were recorded every 4 h at 20× magnification to monitor cell confluence during the desired period for controls and the experimental fraction. Confluence was evaluated using the IncuCyte SX1 software V. 2023A Rev 2 (Sartorius Bioanalytical Instruments, Göttingen, Germany) and expressed as % confluency. Cell death was evaluated after 24 h and 48 h with 10 µM propidium iodide (PI, Sigma Aldrich, P4170-25MG, St. Louis, Missouri, USA) staining for 10 min. Fluorescence images were taken in the IncuCyte SX1 System and cell death was expressed as % red (PI+) confluency/% total confluency.

### 4.7. Assessment of Caspase-3/7 Activity

The cells were seeded in 96-well plates and treated with the vehicle (DMSO) and different concentrations of EAF (7.81–500 µg/mL). All treatment groups were also treated with IncuCyte Caspase-3/7 Green Apoptosis Assay Reagent (cat. 4440, Sartorius) diluted to a final concentration of 5 µM, according to the manufacturer’s instructions. The plates were placed in the IncuCyte SX1, and 4 images were captured every 4 h for 48 h with a 20× objective lens in each well. Using the IncuCyte SX1 software, V. 2023A Rev 2, a fluorescence threshold was established in representative images and applied to all images in the experiment, allowing quantification of fluorescence in all treatment groups.

### 4.8. Cell Apoptosis Analysis

Apoptosis induced by EAF in PANC-1 cells was evaluated via flow cytometry using the Annexin V/7-AAD Apoptosis Detection Kit (cat. 640922, BioLegend) according to the manufacturer’s instructions. PANC-1 cells were cultured in 6-well plates and treated with 7.81, 31.25, 62.5, and 250 µg/mL concentrations of EAF for 24 h. After treatment, cells were washed with PBS, trypsinized, and resuspended in binding buffer. Five microliters of AnnexinV and 7-AAD were added, resulting in a final concentration of 2 µg/mL. Cells were incubated for 15 min in the dark, and the results were immediately analyzed by flow cytometry (BD FACSCanto II; FITC for Annexin V; PerCP for 7-AAD). Data were processed with FlowJo v10. Finally, percentages of different populations of cells are distinguished as nonapoptotic, apoptotic, and necrotic cells.

### 4.9. Cell Proliferation Assay

Antiproliferative effects of different concentrations of EAF were measured using carboxyfluorescein succinimidyl ester (CFSE) staining (cat. C34570, Thermo Fisher Scientific). The quantification of CFSE was performed using flow cytometry and according to the manufacturer’s instructions. PANC-1 cells were plated on 6-well plates and, after 24 h, stained with 5 µM of CFSE. The cells treated with DMSO were considered to be the negative control group, and those treated with different concentrations of EAF for 48 h (7.81, 31.25, 62.5, 250, and 500 µg/mL) were considered to be the experimental groups. After treatment, cells were washed with PBS, trypsinized, and centrifuged. Subsequently, the pellets were resuspended in PBS with 3% FBS for immediate analysis in a BD FACS Canto II flow cytometer.

### 4.10. Statistical Analysis

All statistical analyses were performed using GraphPad Prism 8.01 software. All experimental data are shown as the mean  ±  standard deviation (SD) and analyzed using analysis of variance (ANOVA) and Tukey’s post hoc test. Statistical analyses were performed according to the experimental design. *p * <  0.05 was considered to indicate a statistically significant difference.

## 5. Conclusions

The ethyl acetate fraction of *Euphorbia indivisa* exhibited cytotoxic activity against PANC-1 cells and was associated with reduced cell confluence and increased cell death. The observed increase in caspase-3/7 activity, together with apoptosis assay results, supports the involvement of apoptotic processes in the cellular response to this fraction. Additionally, quercetin, gallic acid, β-sitosterol, palmitic acid, and apigenin-8-*C*-glucoside were identified in *E. indivisa* for the first time, expanding the phytochemical knowledge of this species. Although these compounds are known to possess various biological activities, their individual contributions to the effects observed in the ethyl acetate fraction remain to be determined. Overall, these findings support the value of further investigating this species as a potential source of bioactive compounds. Furthermore, additional studies are needed to elucidate the mechanisms of action and establish compound selectivity using appropriate cellular models. Further purification of the active fractions and reevaluation of their biological activity will help clarify their pharmacological potential. To the best of our knowledge, this study represents the first report on the cytotoxic activity of *E. indivisa* against pancreatic cancer cells and its association with apoptotic responses in PANC-1 cells.

## Figures and Tables

**Table 1:** Compounds identified in species of the genus *Euphorbia*.

Species	Part of the Plant	Compounds	Activities	Reference
*E. humifusa*	Whole plant	β-Sitosterol, Quercetin	Antioxidant and antitumor	[39]
*E. hirta*	Aerial parts	β-Sitosterol, palmitic acid	anti-inflammatory, antibacterial	[40,41]
*E. paralias*	Aerial parts	Palmitic acid, Quercetin, β-sitosterol	Anticancer	[42]
*E. tirucalli*	Root, latex, and stem	Quercetin, taraxasterol	Antibacterial, anti-inflammatory, antioxidant, and anticancer	[43,44]
*E. greenwayi*	Aerial parts	Lupeol	Cytotoxic and cicatrizant	[45]

## Data Availability

All data generated and analyzed in the current study are included in this article.

## Supplementary Materials

The following supporting information can be downloaded at: https://www.mdpi.com/article/10.3390/ijms27219858/s1.

## Abbreviations

The following abbreviations are used in this manuscript:

AR	Aqueous residue
BuF	Butanol fraction
CC	Column chromatography
CD_3_OD	Deuterated methanol
CDCl_3_	Deuterated chloroform
CFSE	Carboxyfluorescein succinimidyl ester
DCF	Dichloromethane fraction
DMSO-*d*_6_	Deuterated dimethyl sulfoxide
EAF	Ethyl acetate fraction
FBS	Fetal bovine serum
GC-MS	Gas chromatography–mass spectrometry
HaCaT	Immortalized keratinocyte cell line
HAE	Hydroalcoholic extract
IC_50_	Half-maximum inhibitory concentration
MCF-7	Breast cancer cell line
NMR	Nuclear magnetic resonance
PANC-1	Human pancreatic ductal adenocarcinoma cell line
PDX	Podophyllotoxin
PI	Propidium iodide
SI	Selectivity index
SiHa	Human cervical cancer cell line
SRB	Sulforhodamine B
TLC	Thin-layer chromatography
