# Supplementary material for: Evaluation of the Cytotoxic and Apoptotic Activities and the Phytochemical Profile of Euphorbia indivisa (Engelm) Tidestr
Source: Int J Mol Sci. 2026 Nov 3;27(21):9858. doi: 10.3390/ijms27219858 (PMC13763397; doi:10.3390/ijms27219858)
Supplement: Supplementary file 1 [file ijms-27-09858-s001.zip › ijms-4545690-supplementary.pdf]

Evaluation of the cytotoxic and apoptotic activities, and the phytochemical profile of *Euphorbia indivisa* (Engelm) Tidestr.

Mónica I. Ramos-Adame [1,a], Paola Maycotte [2], Fabiola L. Sarmiento-Salinas [2,3], Mónica Morales-Aguilar [4], Alexandre T. Cardoso-Taketa [4] and A. Berenice Aguilar-Guadarrama [1,*]

Appendix A

Appendix A.1

Table S1. IC_{50} of the extract (HAE) and fractions (DCF, EAF, BuF, and AR) of *E. indivisa* against breast cancer (MCF-7), pancreatic cancer (PANC-1), cervical cancer (SiHa), and immortalized human keratinocyte cells (HaCaT).

**MCF-7**	**HAE**	**DCF**	**EAF**	**BuF**	**AR**
**Log(inhibitor) vs. normalized response**					
$LogIC_{50}$	2.351	2.401	2.069	2.270	2.345
IC_{50}	224.2	251.7	117.2	186.4	221.1
**95% CI (profile likelihood)**					
$LogIC_{50}$	2.240 to 2.467	2.281 to 2.527	1.946 to 2.195	2.171 to 2.373	2.249 to 2.443
IC_{50}	173.6 to 292.8	191.1 to 336.3	88.38 to 156.6	148.1 to 236.1	177.6 to 277.2
**Goodness of Fit**					
Degrees of Freedom	62	62	62	62	62
R squared	0.6802	0.7063	0.7183	0.7637	0.7611
Sum of Squares	20138	28284	29635	19061	15304
Sy.x	18.02	21.36	21.86	17.53	15.71

**PANC-1**	**HAE**	**DCF**	**EAF**	**Bu**	**AR**
**Log(inhibitor) vs. normalized response**					
$LogIC_{50}$	1.866	2.095	1.419	1.830	1.923
IC_{50}	73.52	124.6	26.27	67.59	83.66
**95% CI (profile likelihood)**					
$LogIC_{50}$	1.806 to 1.927	2.022 to 2.170	1.365 to 1.473	1.779 to 1.880	1.870 to 1.975
IC_{50}	63.99 to 84.52	105.2 to 147.8	23.18 to 29.74	60.17 to 75.94	74.10 to 94.49
**Goodness of Fit**					
Degrees of Freedom	83	83	83	83	83
R squared	0.8693	0.7483	0.8342	0.8952	0.8900
Sum of Squares	12782	16250	8127	8242	9213
Sy.x	12.41	13.99	9.895	9.965	10.54

SiHa	HAE	DCF	EAF	BuF	AR
**Log(inhibitor) vs. normalized response**					
$LogIC_{50}$	2.145	2.331	2.101	1.999	2.266
IC_{50}	139.6	214.4	126.2	99.88	184.5
**95% CI (profile likelihood)**					
$LogIC_{50}$	2.051 to 2.241	2.231 to 2.435	1.981 to 2.224	1.908 to 2.092	2.154 to 2.381
IC_{50}	112.4 to 174.2	170.1 to 272.3	95.74 to 167.5	80.92 to 123.7	142.7 to 240.5
**Goodness of Fit**					
Degrees of Freedom	62	62	62	62	62
R squared	0.7029	0.7673	0.7066	0.7734	0.6502
Sum of Squares	27207	17543	25117	14685	19530
Sy.x	18.11	17.10	20.13	15.39	17.75

HaCaT	HAE	DCF	EAF	BuF	AR
**Log(inhibitor) vs. normalized response**					
$LogIC_{50}$	2.354	2.138	2.191	2.356	2.425
IC_{50}	226.1	137.5	155.2	226.8	266.3
**95% CI (profile likelihood)**					
$LogIC_{50}$	2.269 to 2.442	2.038 to 2.240	2.096 to 2.288	2.264 to 2.449	2.353 to 2.499
IC_{50}	185.8 to 276.6	109.2 to 173.9	124.8 to 193.9	183.9 to 281.4	225.7 to 315.5
**Goodness of Fit**					
Degrees of Freedom	83	83	62	62	62
R squared	0.7561	0.7314	0.8160	0.7873	0.8501
Sum of Squares	25658	38321	14522	16209	8118
Sy.x	17.58	21.49	16.25	16.17	11.63

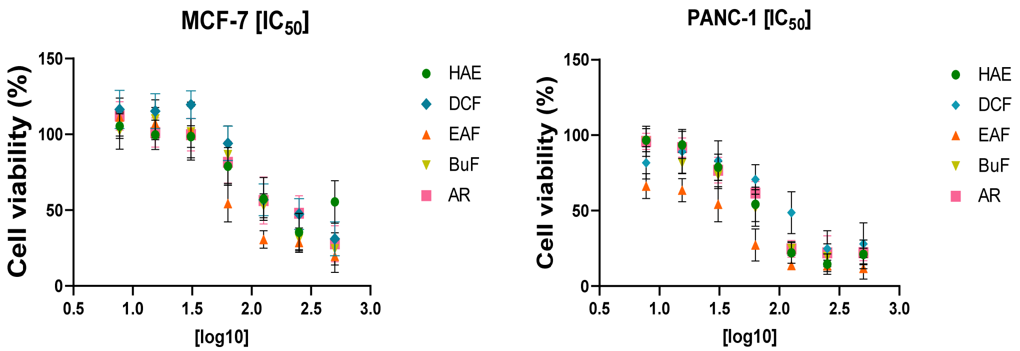

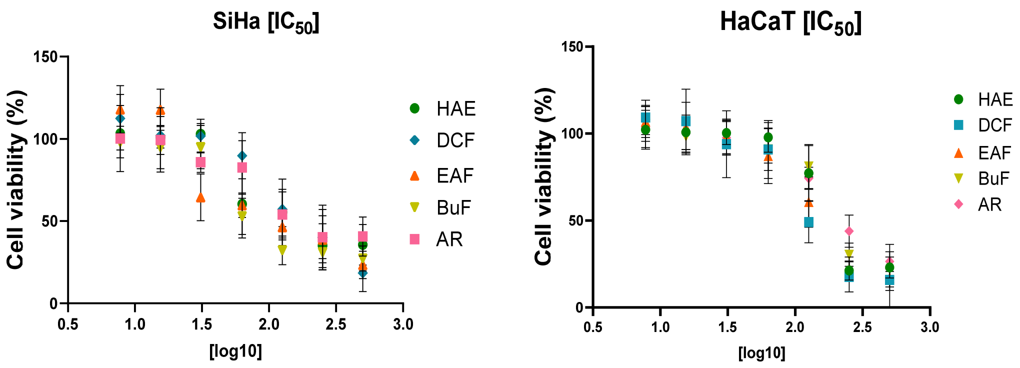

Table S1.1 IC_{50} of podophyllotoxin against breast cancer (MCF-7), pancreatic cancer (PANC-1), cervical cancer (SiHa), and immortalized human keratinocyte cells (HaCaT).

MCF-7	MCF-7	PANC-1	SiHa	HaCaT
**Log(inhibitor) vs. normalized response**				
LogIC50	0.4708	0.4065	1.108	0.2362
IC50	2.837	2.570	12.82	1.722
**95% CI (profile likelihood)**				
LogIC50	0.1347 to 0.7704	0.1886 to 0.6019	0.8639 to 1.360	0.04787 to 0.4163
IC50	1.194 to 6.002	0.8983 to 6.008	7.309 to 22.93	1.117 to 2.608
**Goodness of Fit**				
Degrees of Freedom	14	14	14	14
R squared	0.1176	0.2531	0.5760	0.8453
Sum of Squares	2631	2671	1535	2756
Sy.x	13.71	13.81	10.47	14.03

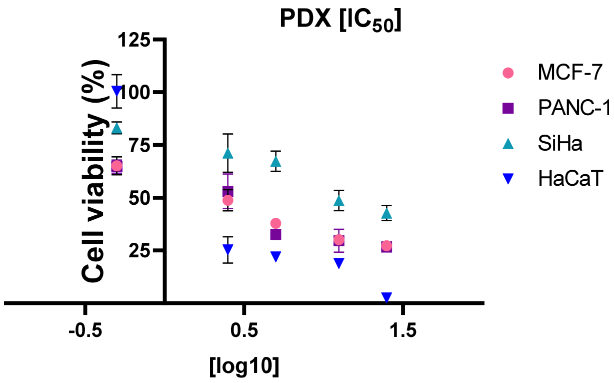

Appendix A.2

Table S2. Selectivity index values of the extract and fractions of *E. indivisa* in cancer cell.

	SI		
**Extract/**	Cell line		
**Fraction**	**MCF-7**	**PANC-1**	**SiHa**
**HAE**	1.00	3.07	1.61
**DCF**	0.54	1.10	0.64
**EAF**	1.32	5.90	1.22
**BuF**	1.21	3.35	2.27
**AR**	1.20	3.18	1.44

Appendix A.3

Figure S1. NMR spectra of the compounds identified in *E. indivisa*

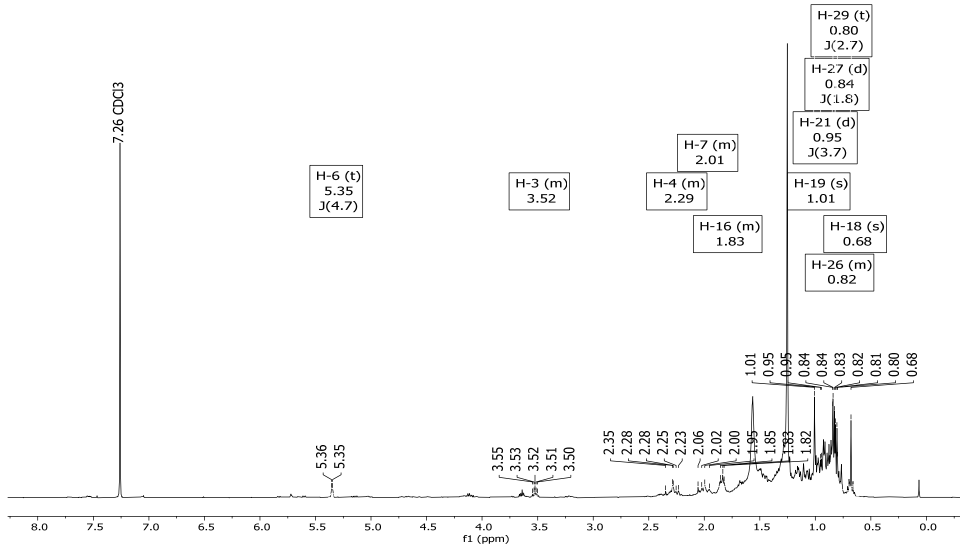

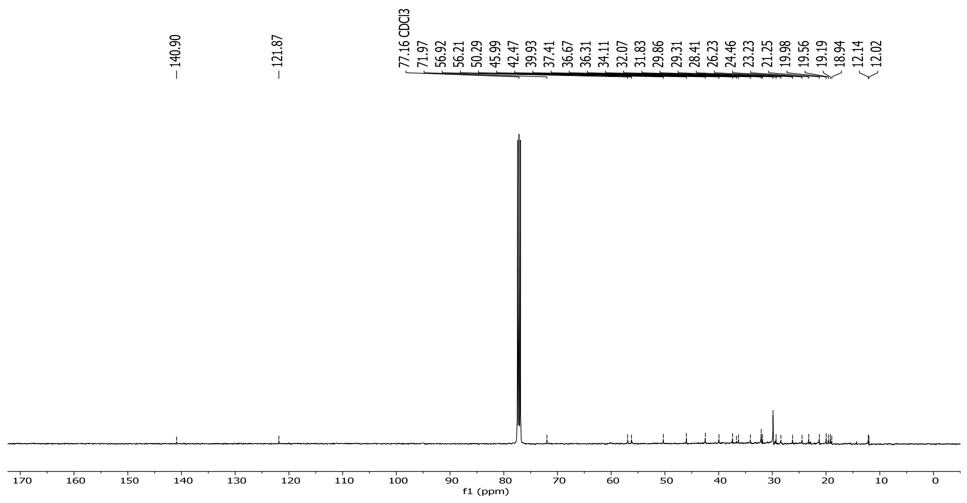

Position	Type	δ (ppm) ^{13}C NMR (125 MHz) ^{1}H (500 MHz), $CDCl_3$	
		^{13}C	^{1}H
**1**	CH_2	37.41	
**2**	CH_2	29.86	
**3**	CH (OH)	71.97	3.52 (m)
**4**	CH_2	42.47	2.29, (m)
**5**	C (=)	140.90	–
**6**	CH (=)	121.87	5.35, (t)
**7**	CH_2	32.07	2.01, (m)
**8**	CH	31.83	
**9**	CH	50.29	
**10**	C	36.67	–
**11**	CH_2	21.25	
**12**	CH_2	39.93	
**13**	C	42.47	–
**14**	CH	56.92	
**15**	CH_2	24.46	

**16**	CH_2	29.31	1.83, (m)
**17**	CH	56.21	
**18**	CH_3	12.02	0.68, (s)
**19**	CH_3	19.56	1.01, (s)
**20**	CH	36.31	
**21**	CH_3	18.94	0.95, (d)
**22**	CH_2	34.11	
**23**	CH_2	26.23	
**24**	CH	45.99	
**25**	CH	28.41	
**26**	CH_3	19.98	0.82, (m)
**27**	CH_3	19.19	0.84, (d, J=1.8)
**28**	CH_2	23.23	
**29**	CH_3	12.14	0.80, (t)

a) ^1H and ^{13}C NMR spectra of β-sitosterol

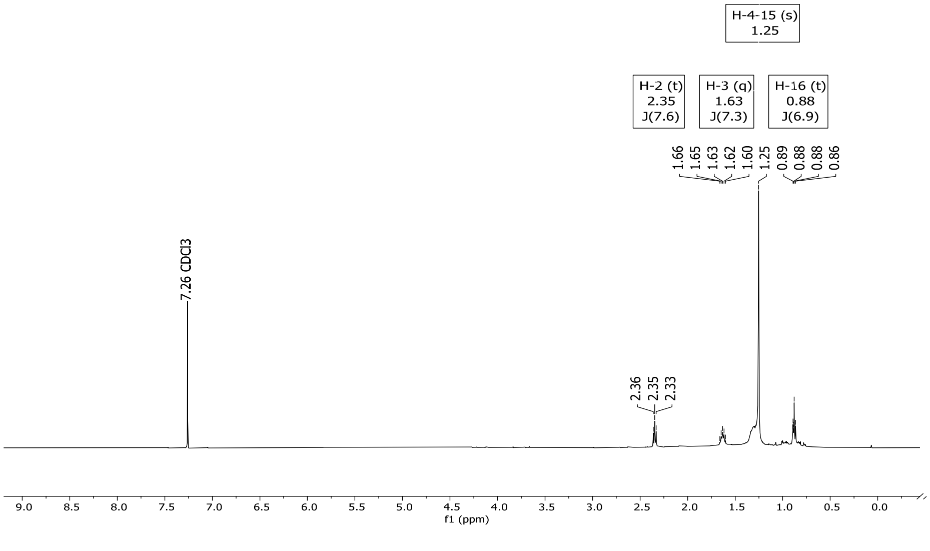

Position	Type	δ (ppm) ^{13}C NMR (125 MHz) ^{1}H (500 MHz), $CDCl_3$
		**^{1}H**
**2**	CH_2	2.35 (t, J=7.6)
**3**	CH_2	1.63 (q)
**4**	CH_2	1.25 (s)
**5**	CH_2	1.25 (s)
**6**	CH_2	1.25 (s)
**7**	CH_2	1.25 (s)
**8**	CH_2	1.25 (s)
**9**	CH_2	1.25 (s)
**10**	CH_2	1.25 (s)
**11**	CH_2	1.25 (s)
**12**	CH_2	1.25 (s)
**13**	CH_2	1.25 (s)
**14**	CH_2	1.25 (s)
**15**	CH_2	1.25 (s)
**16**	CH_3	0.88, (t, J=6.9)

b) ^{1}H NMR spectra of Palmitic acid

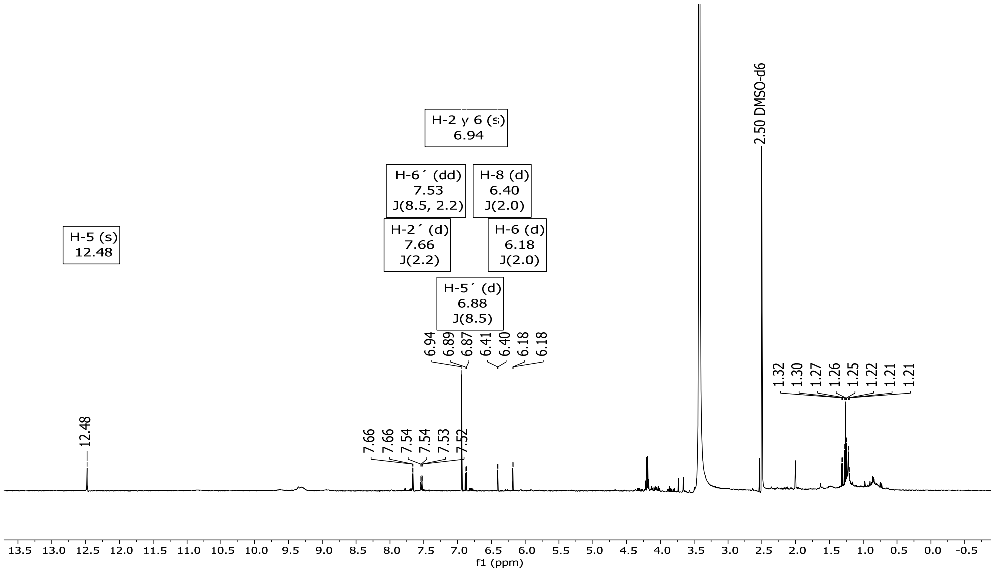

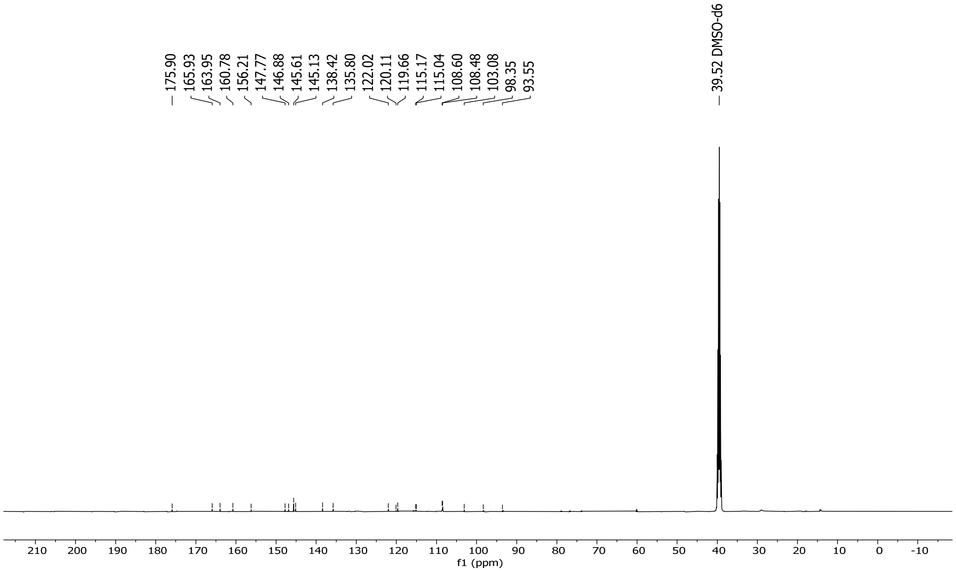

		Quercetin	
**Position**	**Type**	**δ (ppm) ^{13}C NMR (150 MHz) ^{1}H (600 MHz), DMSO-d6**	
		**^{13}C**	**^{1}H**
**2**	C-O	146.88	–
**3**	C-O	138.42	–
**4**	C=O	175.90	–
**5**	C-O	160.78	12.48 (s)
**6**	CH	98.35	6.18 (d, 2.0)

**7**	C-O	163.95	–
**8**	CH	93.55	6.40, (d, 2.0)
**9**	C-O	156.21	–
**10**	C	103.08	–
**1'**	C	122.02	–
**2'**	CH	115.04	7.66 (d, 2.2)
**3'**	C-O	145.13	–
**4'**	C-O	147.77	–
**5'**	CH	115.17	6.88 (d, 8.5)
**6'**	CH	119.66	7.53 (dd, 8.5 y 2.2)

Gallic acid			
**Position**	**Type**	**δ (ppm) ^{13}C NMR (150 MHz) ^{1}H (600 MHz), DMSO-d6**	
		**^{13}C**	**^{1}H**
**1**	C	120.11	–
**2**	CH	108.60	6.94 (s)
**3**	C-O	145.61	–
**4**	C-O	138.42	–
**5**	C-O	145.61	–
**6**	CH	108.48	6.94 (s)
**7**	C=O	165.93	–

c) ^{1}H and ^{13}C NMR spectra of the mixture of Quercetin and Gallic acid.

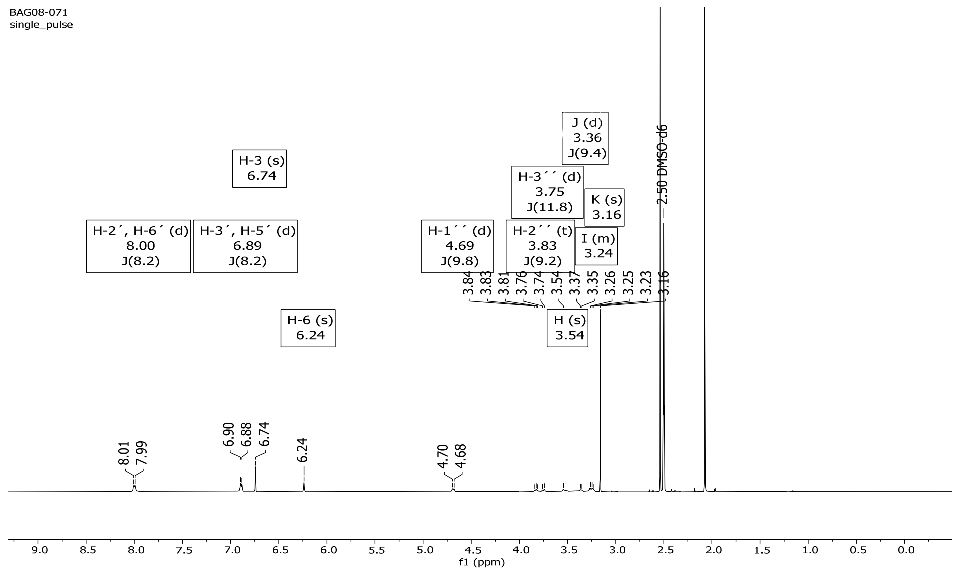

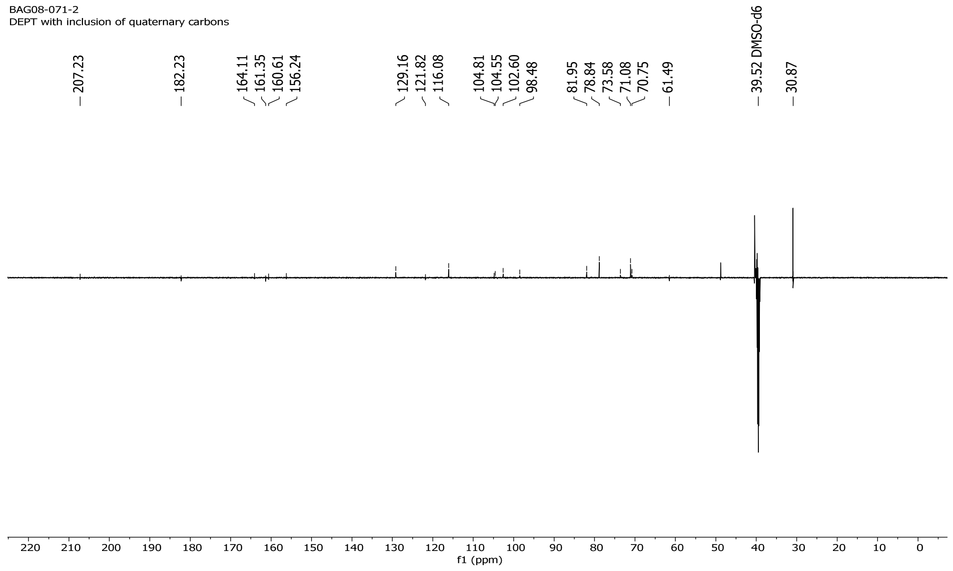

Position	Type	δ (ppm) ^{13}C NMR (150 MHz) ^{1}H (600 MHz), DMSO-d6	
		^{13}C	^{1}H
**2**	C	164.11	
**3**	CH	102.60	6.74 (s)
**4**	C=O	182.23	
**5**	C	160.61	
**6**	CH	98.48	6.24 (s)
**7**	C	161.35	

**8**	C	104.55	
**9**	C	156.24	
**10**	C	104.81	
**1′**	C	121.82	
**2′**	CH	129.16	8.00 (d, J=8.2)
**3′**	CH	116.08	6.89 (d, J=8.2)
**4′**	C	160.61	
**5′**	CH	116.08	6.89 (d, J=8.2)
**6′**	CH	129.16	8.00 (d, J=8.2)
**1′′**	CH	73.58	4.69 (d, J=9.8)
**2′′**	CH	71.08	3.83 (t, J=9.2)
**3′′**	CH	78.84	3.75 (d, J=11.8)
**4′′**	CH	70.75	3.54
**5′′**	CH	81.95	3.36
**6′′**	CH_2	61.49	3.24

d) ^{1}H and DEPTQ spectra of Apigenin-8-*C*-glucoside

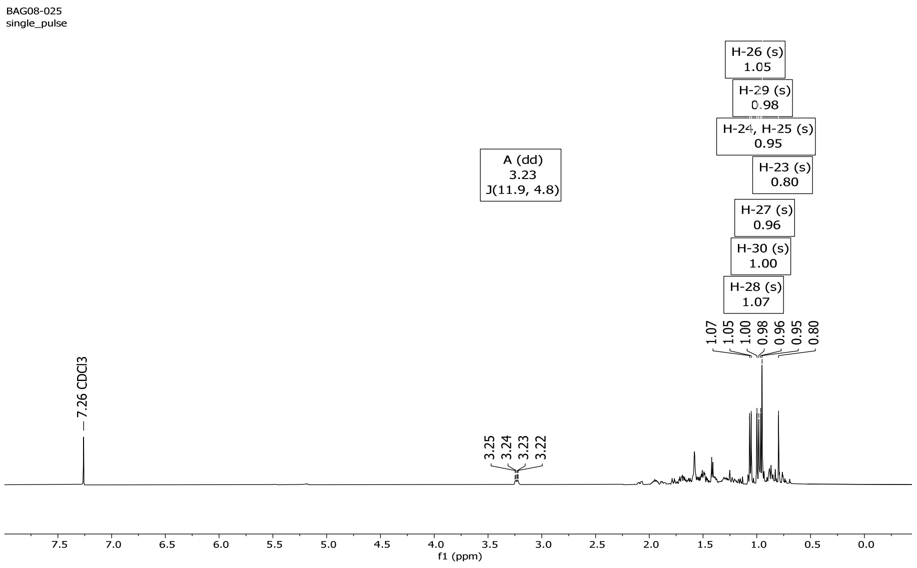

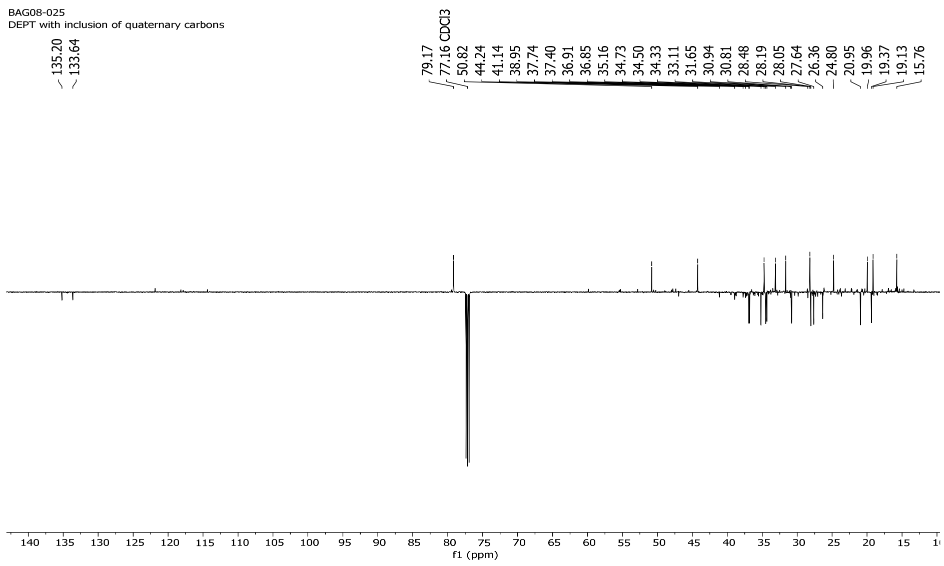

Position	Type	δ (ppm) ^{13}C NMR (125 MHz) ^{1}H (500 MHz), $CDCl_3$	
		^{13}C	^{1}H
**1**	CH_2	34.50	
**2**	CH_2	26.35	
**3**	CH (OH)	79.17	3.23 (dd, *J* = 11.9 y 4.8 Hz)
**4**	C	37.74	
**5**	CH	50.82	
**6**	CH_2	20.95	
**7**	CH_2	28.05	
**8**	C	135.20	
**9**	C	133.64	
**10**	C	37.40	
**11**	CH_2	19.37	
**12**	CH_2	30.81	
**13**	C	38.95	
**14**	C	41.14	
**15**	CH_2	27.64	

**16**	CH_2	35.16	
**17**	C	30.94	
**18**	CH	44.24	
**19**	CH_2	34.33	
**20**	C	28.47	
**21**	CH_2	36.85	
**22**	CH_2	36.91	
**23**	CH_3	28.19	0.80 (s)
**24**	CH_3	15.76	0.95 (s)
**25**	CH_3	19.13	0.95 (s)
**26**	CH_3	19.96	1.05 (s)
**27**	CH_3	24.80	0.97 (s)
**28**	CH_3	31.65	1.07 (s)
**29**	CH_3	33.11	0.98 (s)
**30**	CH_3	34.73	1.00 (s)

e) ^1H and DEPT spectra of Isomultiflorenol

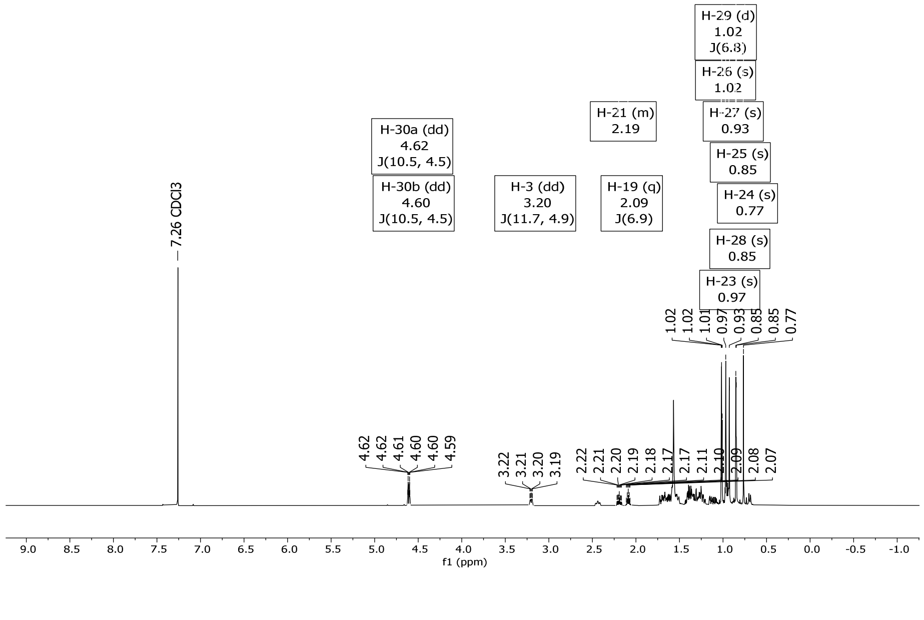

BAG08-027
DEPT with inclusion of quaternary carbons

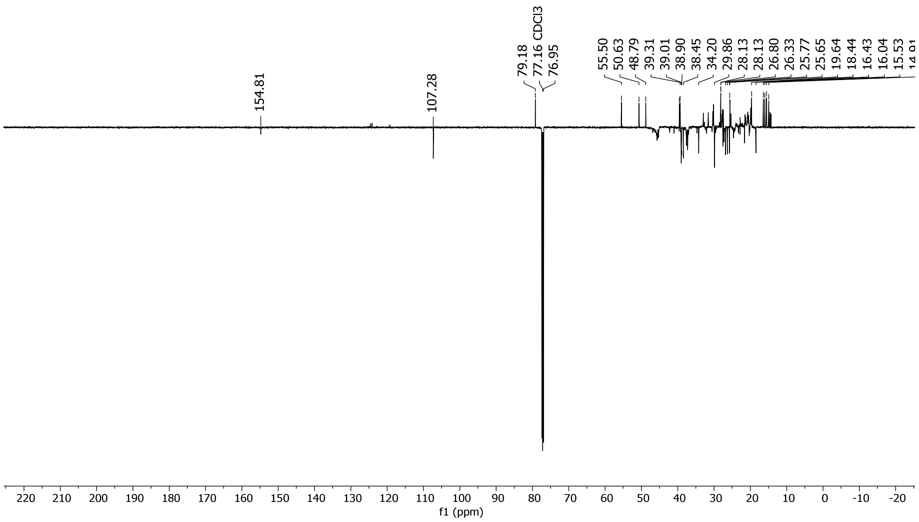

Position	Type	δ (ppm) ^{13}C NMR (150 MHz) ^{1}H (600 MHz), $CDCl_3$	
		^{13}C	^{1}H
**1**	CH_2	38.91	
**2**	CH_2	27.55	
**3**	CH (OH)	79.18	3.20 (dd, J = 11.7 y 4.9)
**4**	C	37.59	
**5**	CH	55.50	
**6**	CH_2	18.45	
**7**	CH_2	34.21	
**8**	C	41.04	
**9**	CH	50.63	
**10**	C	37.25	
**11**	CH_2	21.59	
**12**	CH_2	26.34	
**13**	CH	39.32	
**14**	C	42.19	

**15**	CH_2	26.81	
**16**	CH_2	38.45	
**17**	C	34.69	
**18**	CH	48.79	
**19**	CH	39.53	2.09 (q, J = 6.9 Hz)
**20**	C	154.81	
**21**	CH_2	25.77	2.18 (m); 2.42 (m)
**22**	CH_2	39.02	
**23**	CH_3	28.13	0.97 (s)
**24**	CH_3	16.04	0.77 (s)
**25**	CH_3	15.53	0.85 (s)
**26**	CH_3	16.44	1.02 (s)
**27**	CH_3	14.92	0.93 (s)
**28**	CH_3	19.64	0.85 (s)
**29**	CH_3	25.65	1.02 (d, J = 6.8)
**30**	CH_2	107.28	4.60 (H-b),<br>4.62 (H-a)

f) ^1H and DEPT spectra of Taraxasterol

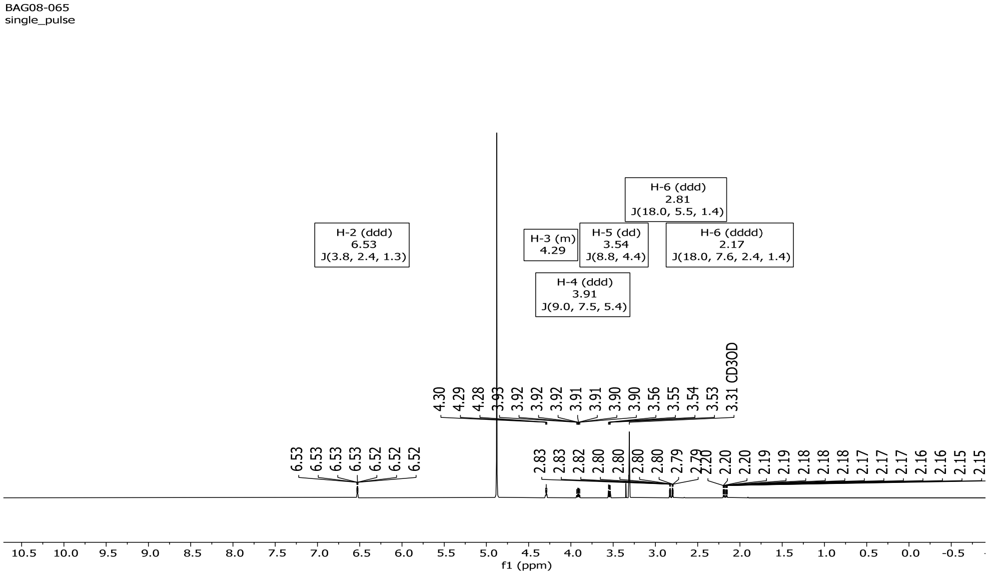

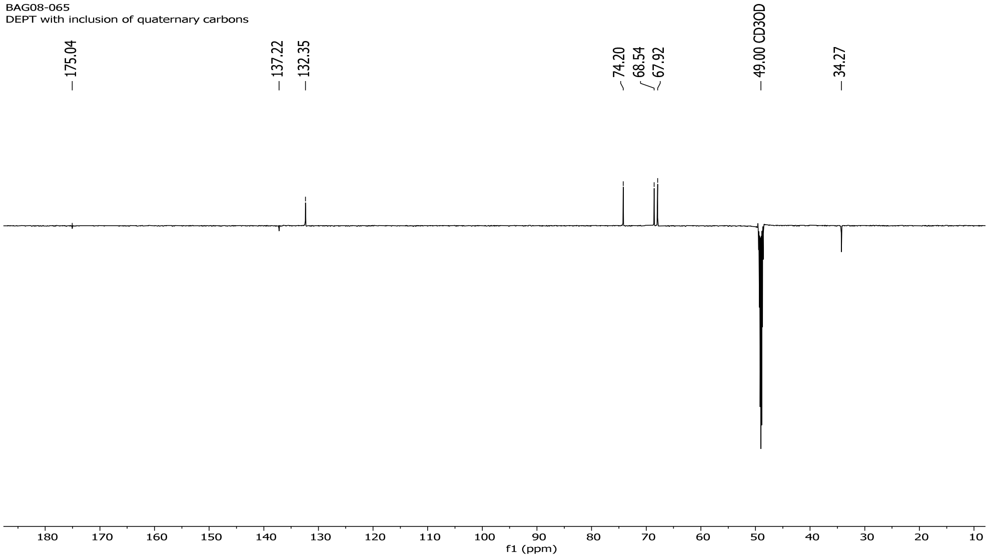

**Position**	**Type**	**δ (ppm) ^{13}C NMR (150 MHz) ^{1}H (600 MHz), CD$_3$OD**	
		^{13}C	^{1}H
**1**	C=C	132.35	–
**2**	C=C	137.22	6.53 (ddd, J= 3.8, 2.4, 1.3)
**3**	C-O	68.54	4.29 (m)
**4**	C-O	74.20	3.91 (ddd, J= 9.0, 7.5, 5.4)
**5**	C-O	67.92	3.54 (dd J= 8.8, 4.4)
**6**	CH$_2$	34.27	2.81 (ddd, J= 18.0, 5.5, 1.4)<br>2.16 (dddd, J=18.0, 7.6, 2.4, 1.4)
**7**	C=O	175.04	–

g) ^{1}H and DEPT spectra of Shikimic acid.

Appendix A.4

Table S3. Compounds identified by GC-MS in aerial parts of *E. indivisa*.

No.	Compounds	MW (g/mol)	MF	Category	Rt (min)	Relative peak area %	Biological Activity	Reference
**2**	Palmitic acid	256.3	$C_{16}H_{32}O_2$	Fatty acid	68.732	1	Antioxidant and antibacterial	[40]
**8**	Phytol	296.3	$C_{20}H_{40}O$	Diterpene	69.553	2.66	Cytotoxic, antimicrobial, antioxidant and anti-inflammatory	[24-26]
**9**	α-Tocospiro A	462.4	$C_{29}H_{50}O_4$	Tocopherol	97.641	4.57	Antioxidant	[27]
**10**	13,27-cycloursan-3-ol	426.4	$C_{30}H_{50}O$	Triterpene	111.855	2.53	Antimicrobial and antifungal	[28]
**11**	α-amyrin	426.4	$C_{30}H_{50}O$	Triterpene	112.092	0.77	Cytotoxic and anti-inflammatory	[29,30]
**12**	β-amyrin	426.4	$C_{30}H_{50}O$	Triterpene	112.131	4.37	Cytotoxic and anti-inflammatory	[29,30]
**13**	Lanosterol	426.4	$C_{30}H_{50}O$	Triterpene	115.271	22.29	Antimicrobial and antioxidant	[31]
**14**	Stigmast-4-en-3-one	412.4	$C_{29}H_{48}O$	Sterol	115.514	6.96	Cytotoxic	[32]
**15**	Lupeol	426.4	$C_{30}H_{50}O$	Triterpene	116.716	6.03	Hypoglycemic, antihyperglycemic, antimicrobial, anticancer, and anti-inflammatory	[33,34]

MW: Molecular weight; MF: Molecular formula; Rt: Retention time.
